# Acutely damaged axons are remyelinated in multiple sclerosis and experimental models of demyelination

**DOI:** 10.1002/glia.23167

**Published:** 2017-05-31

**Authors:** Verena Schultz, Franziska van der Meer, Claudia Wrzos, Uta Scheidt, Erik Bahn, Christine Stadelmann, Wolfgang Brück, Andreas Junker

**Affiliations:** ^1^ Institute of Infection, Immunity and Inflammation, University of Glasgow 120 University Place Glasgow G12 8TA United Kingdom; ^2^ Institute of Neuropathology, University Medical Center Robert‐Koch‐Straße 40 Göttingen D‐37075 Germany; ^3^ Institute of Neuropathology, University Hospital Essen Hufelandstr. 55 Essen D‐45122 Germany

**Keywords:** axonal damage, multiple sclerosis, remyelination

## Abstract

Remyelination is in the center of new therapies for the treatment of multiple sclerosis to resolve and improve disease symptoms and protect axons from further damage. Although remyelination is considered beneficial in the long term, it is not known, whether this is also the case early in lesion formation. Additionally, the precise timing of acute axonal damage and remyelination has not been assessed so far. To shed light onto the interrelation between axons and the myelin sheath during de‐ and remyelination, we employed cuprizone‐ and focal lysolecithin‐induced demyelination and performed time course experiments assessing the evolution of early and late stage remyelination and axonal damage. We observed damaged axons with signs of remyelination after cuprizone diet cessation and lysolecithin injection. Similar observations were made in early multiple sclerosis lesions. To assess the correlation of remyelination and axonal damage in multiple sclerosis lesions, we took advantage of a cohort of patients with early and late stage remyelinated lesions and assessed the number of APP‐ and SMI32‐ positive damaged axons and the density of SMI31‐positive and silver impregnated preserved axons. Early de‐ and remyelinating lesions did not differ with respect to axonal density and axonal damage, but we observed a lower axonal density in late stage demyelinated multiple sclerosis lesions than in remyelinated multiple sclerosis lesions. Our findings suggest that remyelination may not only be protective over a long period of time, but may play an important role in the immediate axonal recuperation after a demyelinating insult.

**Abbreviations**
APP: amyloid precursor protein
EAE: experimental autoimmune encephalomyelitis
NO, nitric oxide
ROS: reactive oxygen species

## 1 INTRODUCTION

Persistent disability and disease progression in multiple sclerosis are tightly connected to neuro‐axonal damage and loss (Simons, Misgeld, & Kerschensteiner, 2014; Singh et al., 2017). Disturbances of axonal transport and metabolism, often visualized by immunohistochemistry for axonally transported proteins, such as amyloid precursor protein (APP) or SMI32, a marker for dephosphorylated neurofilaments of damaged axons, are most abundant in early stages of lesion formation and, according to experimental data, only a proportion of these damaged axons will undergo axonal transection (Sorbara et al., 2014; Nikic et al., 2011; Trapp et al., 1998). A number of inflammatory mediators have been accused to impair axonal function, mainly derived from phagocytes actively stripping off the myelin sheaths in early lesions (Clark et al., 2016; Cunningham, 2013; Lassmann, 2014). Experimentally, NO and ROS, highly reactive lipid soluble mediators, are responsible for an important share of the acute axonal damage observed (Nikic et al., 2011; Sorbara et al., 2014). In addition to therapeutically addressing the plethora of inflammatory mediators present, remyelination of axons is considered as one of the most important endogenous axon‐protective mechanisms. Aside from restoring the conductive properties of the axon, remyelination provides insulation of the axon against a potentially hostile microenvironment and restores the close metabolic interaction with oligodendrocytes (Fünfschilling et al., 2012; Lee et al., 2012; Morrison et al., 2015). The correlation of early remyelination and axonal damage and whether damaged axons can be remyelinated, is unknown so far.

To study the interrelation between demyelination, remyelination, and axonal damage in detail, we employed the mouse model of cuprizone‐induced demyelination and focal lysolecithin‐induced demyelination and performed tight time course experiments assessing the evolution of remyelination and acute axonal damage. We found that the density of APP‐positive axons gradually declined after removing cuprizone from the diet and during the phase of lesion repair after lysolecithin injection, indicating an overall beneficial role of remyelination. In addition, APP‐positive axonal profiles enwrapped by myelin sheaths were found in early remyelinating multiple sclerosis lesions as well as during remyelination in our experimental models. In the present work, we took also advantage of a cohort of patients with early and late stage remyelinated lesions and assessed the density of damaged and preserved axons. No significant difference in the density of preserved and damaged axons could be observed between remyelinating and demyelinating multiple sclerosis lesions early in lesion formation. However, we could demonstrate that in late stage disease, the amount of preserved axons was significantly higher in remyelinated than in demyelinated multiple sclerosis lesions.

Our findings suggest that the re‐establishment of a myelin sheath does not depend on a functional axonal transport and intact axonal metabolism and might even aid the recuperation of damaged axons. Thus, our data support a protective role of remyelination in early as well as late stage lesion development.

## 2 MATERIALS AND METHODS

### 2.1 Multiple sclerosis tissue

In this study formalin‐fixed and paraffin‐embedded biopsy and autopsy CNS tissue of patients with multiple sclerosis was used, which was collected at the Institute of Neuropathology at the University Medical Centre Göttingen (Supplementary Table 1 and 2). Lesions were assessed by A.J. and C.S. according to criteria described elsewhere (Brück et al., 1995, 1994; Kutzelnigg et al., 2005; Lassmann, 2011; Lucchinetti et al., 2000; Schuh et al., 2014). The study was performed in accordance with national and local ethics regulations and approved by the UMG ethics committee.

### 2.2 Animals

For all *in vivo* experiments 6 to 7‐week‐old male C57BL/6J mice and adult female Lewis rats (200–220 g) were purchased from Charles River (Sulzfeld, Germany). Up to 6 animals were housed together on a 12/12 h light/dark cycle and had access to food and water *ad libitum*. Animal experiments were conducted in accordance with the European Communities Council Directive of November 24^th^, 1986 (86/EEC) and were approved by the Government of Lower Saxony, Germany.

### 2.3 Cuprizone mouse model

About 7 to 8‐week‐old male C57BL/6J mice were fed for 6 weeks with the cupper chelator cuprizone (0.25% in normal chow) to induce demyelination in the brain. After cuprizone withdrawal mice were fed with normal diet to analyze remyelination. During de‐ and remyelination, that is after 6 weeks of cuprizone treatment and 2, 3, 4, 5, 6, 7, 14 and 21 days after cuprizone diet cessation, brains of mice were harvested and processed for analysis by histochemistry and immunohistochemistry. Untreated, age‐matched mice were used as a control. Mouse brain tissue was fixed with 4% paraformaldehyde (PFA) and embedded into paraffin. For histological evaluation 1 µm coronal sections were used.

### 2.4 Focal lysolecithin‐induced demyelination

Adult Lewis rats (200–220 g) were anaesthetized by intraperitoneal injection of ketamine/xylazine. The scalp was opened and the animal was mounted on a stereotactic device. A small hole (Ø 2 mm) was drilled 2 mm lateral and 1 mm caudal to the bregma. One µl of 0.5% lysolecithin (Sigma‐Aldrich) diluted in sterile PBS was slowly (3 min) injected into the corpus callosum (2.5 mm depth) using a fine calibrated glass capillary. Afterwards the capillary was removed and the skin was sutured. Animals were perfused 3, 6, 12, and 20 days postinjection for histochemical and immunohistochemical analysis.

### 2.5 Histology

Bielschowsky silver impregnation and Luxol fast blue periodic acid Schiff (LFB‐PAS) staining were performed according to standard procedures. Primary antibodies utilized for immunohistochemistry were against injured axons (APP, 1:2,000, clone 22C11, Chemicon) and nonphosphorylated neurofilaments (SMI32, 1:1,000, Covance, Princeton, NJ, USA), healthy phosphorylated neurofilaments/axons (SMI31, 1:10,000, Covance, Princeton, NJ, USA), myelin basic protein (MBP, 1:2,000, Dako), activated microglia (Mac3, also known as Lamp2, 1:200, clone M3/84, BD Pharmingen), glial fibrillary acidic protein (GFAP, 1:1,000, Dako), and foamy monocytes and macrophages (CD68, 1:5,000, clone KiM1P). Biotinylated secondary antibodies (GE Healthcare, Jackson ImmunoResearch and DCS Innovative diagnostic system), peroxidase conjugated avidin and DAB (Sigma‐Aldrich) were used for immunohistochemistry. Fluorescence labeled secondary antibodies (Cy3‐conjugated goat anti‐mouse IgG, 1:200, Jackson ImmunoResearch and Alexa488‐conjugated goat anti‐rabbit IgG, 1:200, Molecular Probes, Life technologies) were used for fluorescence immunohistochemistry.

### 2.6 Morphometry and data acquisition

The level of demyelination of the corpus callosum after cuprizone treatment was assessed in LFB‐PAS stained and MBP immunostained sections by a semi‐quantitative score: no demyelination (0), 0–33% demyelination (1), 33%–66% demyelination (2), and more than 66% demyelination (3) (Hiremath et al., 1998). APP^+^ and SMI32^+^ axons and the number of glial cells and infiltrating phagocytes (Mac3^+^, KiM1P^+^, GFAP^+^ cells) were determined in the corpus callosum or in the lesion area at 400× with an ocular counting grid (light microscope BX41 and ocular counting grid WHSZ 10X‐H, both Olympus). To evaluate acute axonal damage in the context of early remyelination, fluorescence double immunohistochemistry (APP and MBP) was analyzed by confocal microscopy (laser scanning confocal microscope FluoView FV1000 (Olympus)). Therefore, 30 randomly selected visual fields with at least one APP^+^ axon were measured per corpus callosum at 400x with 7% zoom. The degree of association of APP with MBP (myelinated APP^+^ axons) was assessed by a qualitative evaluation using the software FV10‐ASW 4.0 viewer. The data were calculated as percent of all assessed APP^+^ axons per corpus callosum.

The acute axonal damage in early stage demyelinating and remyelinating multiple sclerosis lesions was evaluated by fluorescence double immunohistochemistry (APP or SMI32 with MBP) and confocal microscopy. For qualitative analysis, 10 randomly selected visual fields with at least one APP^+^ spheroid were analyzed.

Axonal density within the lesions was determined in sections stained with SMI31 and Bielschowsky silver impregnation by using an axonal counting grid with 25 cross‐points at a magnification of 1000× under oil immersion. The number of axons that crossed a grid line was counted as a fraction of the total number of cross‐points. The number of axons crossing the points in normal appearing white matter was set to 100%. The degree of axon reduction in the lesion is given as the percentage of axon density compared with normal appearing white matter.

### 2.7 Imaging

All images were acquired using either the Fluorescence microscope BX51 (Olympus) and Camera DP71/XM10 (Olympus) or the Laser scanning confocal microscope FluoView FV1000 (Olympus). They are presented as captured; only white balance was applied to light microscopical images.

### 2.8 Statistical analysis

Statistics were calculated using the GraphPad Prism 5.01 software. The Kolmogorov–Smirnov test was carried out to test the data for normal distribution. To compare more than two groups, data were analyzed by one‐way ANOVA for parametric data (Bonferroni's Multiple Comparison test). For analysis of nonparametric data the Kruskal‐Wallis test was used (Dunn's Multiple Comparison test). For comparison of two groups the paired or unpaired *t* test was used for parametric data and the Mann‐Whitney test was used for nonparametric data. A *p*‐value of <0.05 was considered as statistically significant.

## 3 RESULTS

### 3.1 Newly remyelinated axons were accompanied by diminished axonal damage in the presence of microglial activation in the cuprizone mouse model

**Figure 1:**
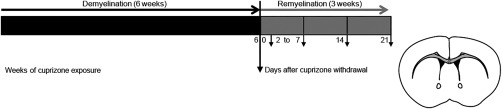
Design of the cuprizone experiments to investigate the timing and relation of acute axonal damage and remyelination. 7 to 8‐week‐old male C57BL/6J mice were fed for 6 weeks with a 0.25% cuprizone diet to induce demyelination in the corpus callosum. Demyelinated lesions remyelinate after cuprizone withdrawal. To study early and late remyelination, CNS tissue was harvested from days 2 to 7, and on days 14 and 21 after cuprizone diet cessation

To elucidate the occurrence of axonal damage over time during the formation of new myelin sheaths, we performed tight time course experiments in the cuprizone mouse model, an animal model for de‐ and remyelination (Figure 1).

**Figure 2:**
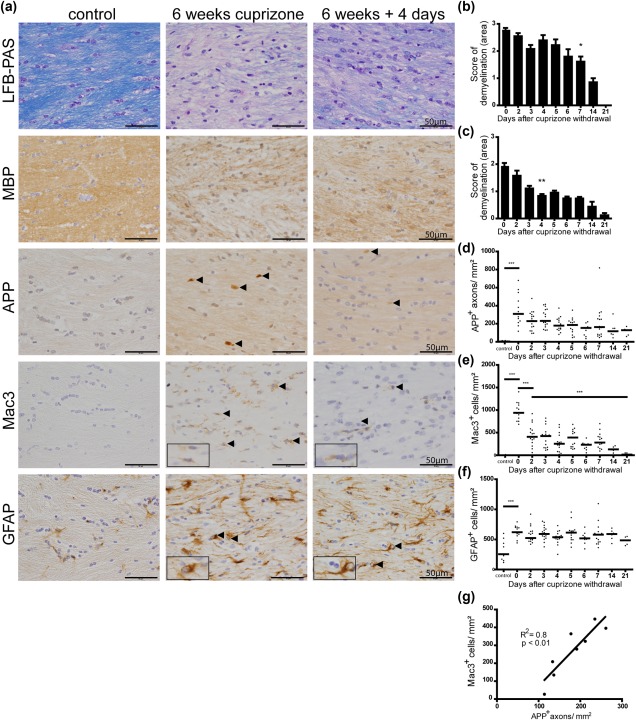
Comprehensive assessment of white matter pathology early and late after cuprizone‐induced demyelination. (a) Representative images of the medial corpus callosum of control mice and mice after cuprizone challenge for 6 weeks and after 4 days of recovery. Myelin assessment by LFB‐PAS histochemistry and MBP immunohistochemistry (IHC) indicated an almost complete demyelination of the corpus callosum after 6 weeks of cuprizone feeding. Significant remyelination was observed 4 days after diet cessation. Acute axonal damage (APP^+^ axons) decreased during remyelination in the cuprizone model. Microglial cells were activated during demyelination. The number of Mac3^+^ activated microglia declined during remyelination in comparison to 6 weeks of demyelination, while the density of reactive astrocytes (GFAP^+^ cells) did not change during the period of remyelination observed here. (b, c) The demyelinated area in the corpus callosum was reduced during remyelination as judged by semi‐quantitative evaluation of LFB‐PAS and MBP IHC. The demyelinated area was significantly reduced after 4 and 7 days (*p < 0.05; **p < 0.01; *n* = 5‐17; mean ± SEM). (d) Acute axonal damage was observed after cuprizone‐induced demyelination in the corpus callosum and was decreased already after 2 days of recovery. The density of APP^+^ axons decreased continuously during remyelination (*p < 0.05; ***p < 0.001; *n* = 5‐17; median). (e) Three days after cuprizone withdrawal the number of activated microglia was significantly diminished and almost absent after 3 weeks of remyelination (***p < 0.001; *n* = 5‐17; mean ± SEM). (f) The evaluation of immunoreactivity for GFAP revealed a persistent activation of astrocytes. (g) The activation of microglia correlated with the amount of damaged axons during recovery after cuprizone diet ingestion (*p*= 0.0028; R^2^= 0.7981; *n* = 8; mean)

To determine the time course of remyelination the content of myelin was analyzed by histochemistry and immunohistochemistry. LFB‐PAS histochemistry and myelin basic protein immunoreactivity revealed an almost complete demyelination of the corpus callosum after 6 weeks of cuprizone treatment (Figure 2a–c). Significant remyelination was observed 4 days after diet cessation. After 1 week of cuprizone withdrawal a major proportion of the corpus callosum was remyelinated, which was almost completed after 3 weeks of recovery (Figure 2b, c).

To investigate the interrelationship of newly formed myelin and acute axonal damage, immunohistochemistry for APP was performed. APP is undetectable in healthy axons, but accumulates in injured axons with disturbed anterograde axonal transport. APP immunohistochemistry revealed swollen axons and axonal spheroids after 6 weeks of cuprizone treatment (median 309.1 APP^+^ axons/mm^2^) and during early (2 days, median 229.5 APP^+^ axons/mm^2^) and long standing (3 weeks, median 129.2 APP^+^ axons/mm^2^) remyelination. The number of APP^+^ axons decreased gradually during remyelination, with a significant difference at day 6 after diet cessation compared with 6 weeks of demyelination (cuprizone feeding), and a few were still present after 3 weeks of recovery (Figure 2a, d).

To assess whether the axons experienced damage because of demyelination, the activation of microglia or the reactivation of astrocytes, we determined the microgliosis and astrogliosis by immunoreactivity for GFAP and Mac3. Many activated microglia (975.9 ± 56.5 cells/mm^2^) and reactive astrocytes (629.3 ± 27.4 cells/mm^2^) were present after 6 weeks of cuprizone treatment (Figure 2a, e, and f). The number of Mac3^+^ cells was significantly diminished 2 days after cuprizone withdrawal. Mac3^+^ cells were almost absent after 3 weeks of recovery (26.76 ± 9.9 cells/mm^2^) (Figure 2a, e). Immunohistochemistry for GFAP displayed a high number of reactive astrocytes in the demyelinated and remyelinated corpus callosum (3 weeks recovery: 478.0 ± 31.07 cells/mm^2^) (Figure 2a, f). Thus, the number of acutely damaged axons decreased during early remyelination in the presence of diminishing microglial activation. Microglial activation was found to strongly correlate with axonal damage, and thus may play a role in its formation (*R* ^2^= 0.7981, *p* = 0.0028) (Figure 2g).

### 3.2 Axons with disturbed axonal transport were remyelinated after cuprizone withdrawal and after lysolecithin‐induced demyelination

**Figure 3:**
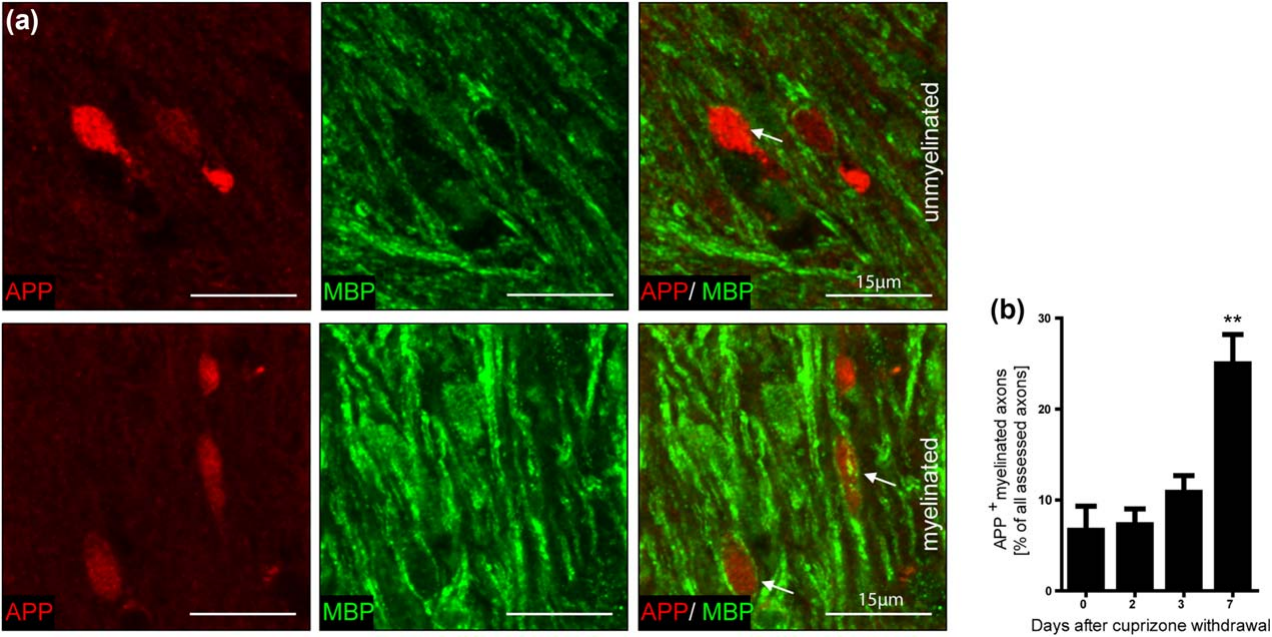
The percentage of myelinated damaged axons increased during remyelination. (a) Unmyelinated and myelinated APP^+^ axons were observed in the corpus callosum of mice after demyelination and during remyelination. A representative image of the corpus callosum 3 and 4 days after withdrawal of cuprizone is shown. (b) APP^+^ axons were analyzed in 30 randomly selected areas with at least one APP^+^ axon (approx. 50 axons) in the corpus callosum of mice by confocal microscopy. After 1 week of recovery the number of myelinated APP^+^ axons increased significantly indicating remyelination of damaged axons (**p < 0.01; *n* = 3‐7; mean ± SEM)

Nikic and colleagues reported that axonal damage is reversible and does not lead to axonal degeneration in all cases (Nikic et al., 2011). To determine whether injured axons can be remyelinated, which would point to a recuperative role of the newly established myelin sheath, a comprehensive confocal microscopy investigation was performed. Double immunostaining of APP and MBP revealed two subpopulations of APP^+^ axons in the corpus callosum of cuprizone‐treated mice (Figure 3a): unmyelinated and myelinated. The percentage of myelinated APP^+^ axons increased significantly during the first 7 days of recovery (from 6.5% to 25%) (Figure 3b). Because of the decrease of the amount of acutely damaged axons and the increase of the number of myelinated, acutely damaged axons over time, it is highly likely that acutely damaged axons gained a new myelin sheath, that were remyelinated.

**Figure 4:**
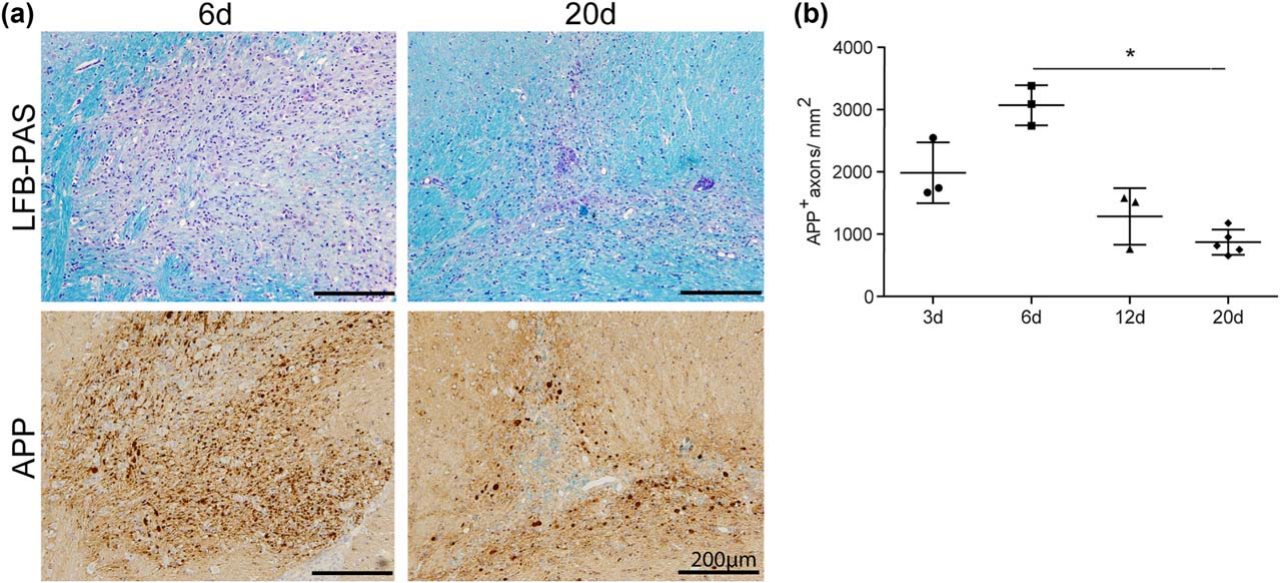
Significant reduction of acute axonal damage during lesion repair in lysolecithin‐induced demyelination. (a) Representative images of lysolecithin‐induced lesions in the rat corpus callosum on days 6 and 20 postinjection. LFB‐PAS histochemistry revealed almost complete demyelination after 6 days and almost complete remyelination after 20 days. APP^+^ axons were observed in lysolecithin‐induced lesions. (b) The number of APP^+^ axons in the lesions increased until day 6 and showed a significant decrease on day 20 after lysolecithin injection (*p<0.05; *n* = 3‐5; mean ± SD)

**Figure 5:**
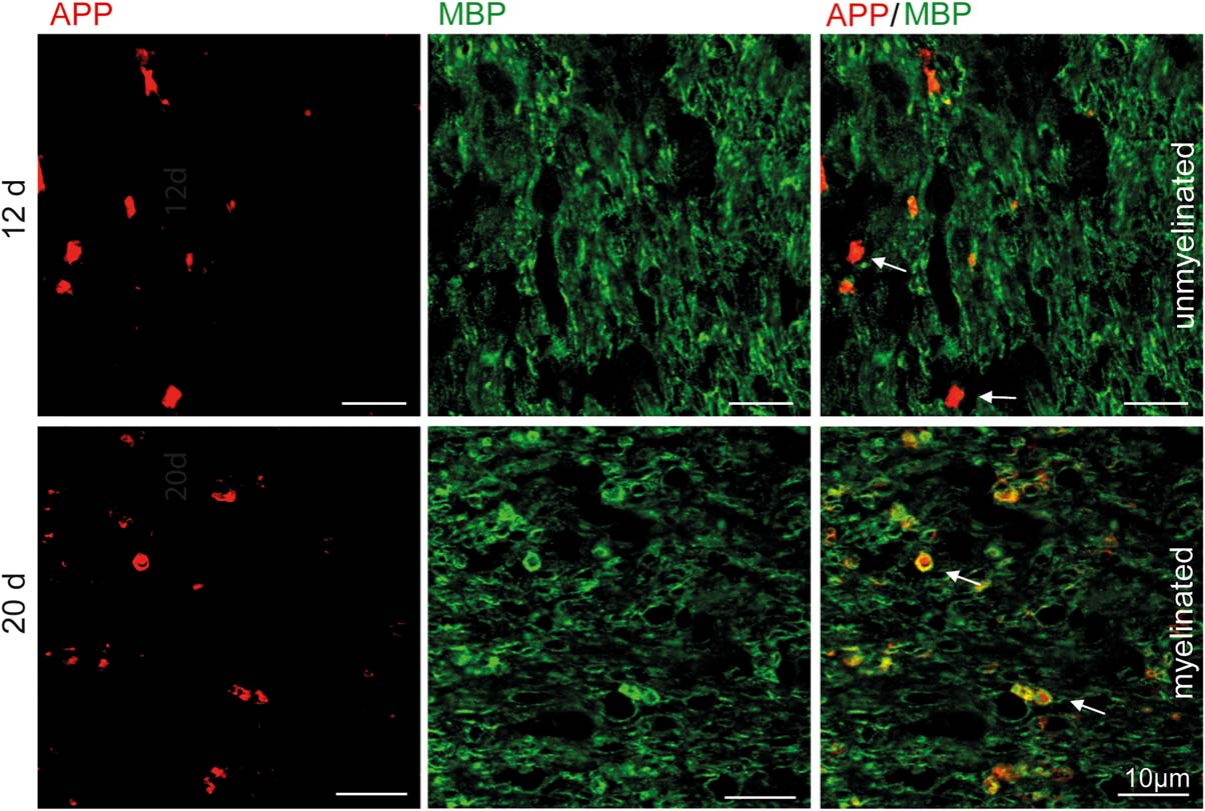
APP^+^ axons are remyelinated in lysolecithin‐induced lesions. Unmyelinated and myelinated APP^+^ axons were detected in lysolecithin‐induced lesions during lesion repair. A representative image 12 and 20 days after lysolecithin injection is shown

To substantiate that the observation that damaged axons undergo remyelination during lesion repair is not restricted to the cuprizone mouse model, we performed focal lysolecithin‐induced demyelination in Lewis rats. In the lysolecithin model, induced by a membrane‐dissolving detergent, axonal damage is important. In contrast to cuprizone‐induced demyelination, lysolecithin‐induced lesions are accompanied by recruitment of peripheral immune cells. Spontaneous remyelination starts a few days after lysolecithin injection. We performed histo‐ and immunohistochemical studies on time course experiments of lysolecithin‐induced lesions. LFB‐PAS histochemistry revealed almost completely demyelinated lesions in the corpus callosum 3 to 6 days after injection, while almost complete remyelination was seen after 20 days (Figure 4a). The number of APP^+^ axons increased during the first 6 days after lysolecithin injection and decreased during the phase of lesion repair, with a significant difference between days 6 and 20 postinjection (from 3070 ± 320.4 to 870.9 ± 220.9 APP^+^ axons/mm^2^) (Figure 4a, b). Double labelling with APP and MBP revealed that also in lysolecithin‐induced demyelination, APP‐positive axons with transport disturbance become remyelinated during lesion repair (Figure 5).

### 3.3 Myelinated damaged axons were observed in early stage multiple sclerosis lesions

**Figure 6:**
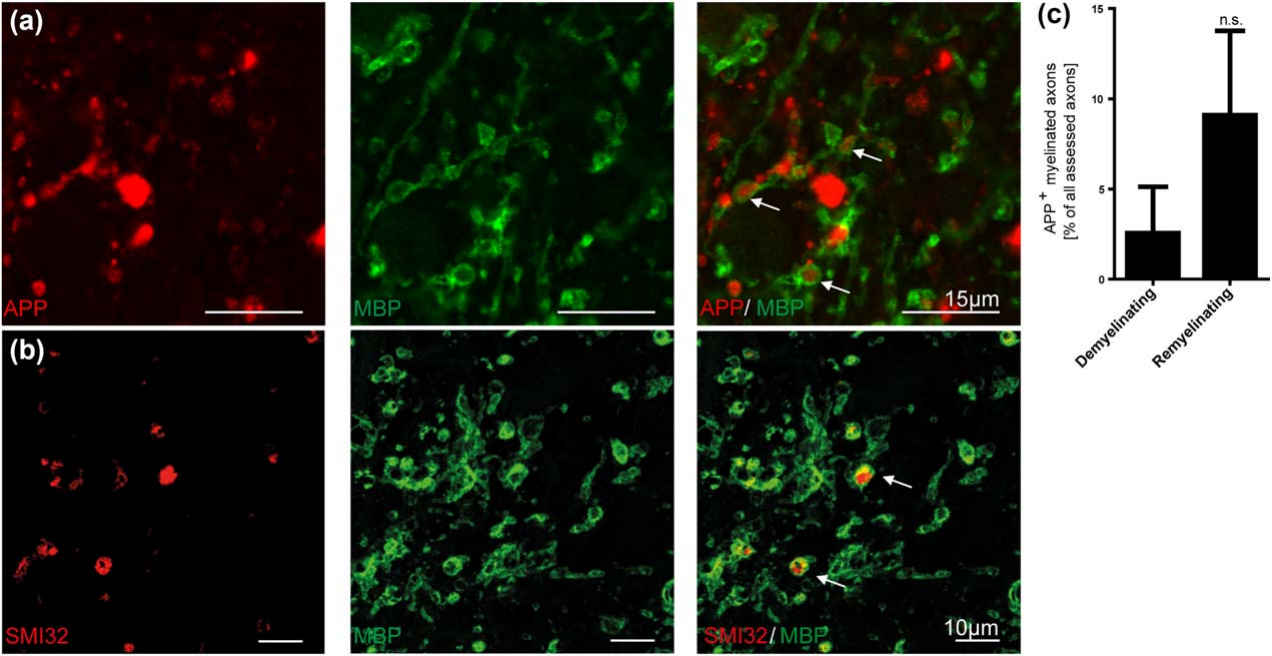
Myelinated APP^+^ and SMI32^+^ axons were found in early remyelinating multiple sclerosis lesions. (a, b) Representative confocal image of myelinated APP^+^ and SMI32^+^ axons (arrows) in an early remyelinating multiple sclerosis lesion. (c) A higher number of myelinated axons with axonal transport disturbance were observed in remyelinating compared with demyelinating multiple sclerosis lesions. This finding suggests that damaged axons in multiple sclerosis lesions are indeed capable of gaining a new myelin sheath (*n* = 3, mean ± SEM)

To verify whether early stage multiple sclerosis lesions exhibit, as seen in the cuprizone and lysolecithin models, (re)myelinated damaged axons, a confocal analysis of demyelinating and remyelinating multiple sclerosis lesions was performed. We found nonmyelinated and myelinated injured axons by double immunohistochemistry for APP or SMI32 and MBP in both lesion types (Figure 6a, b). Although more myelinated APP^+^ damaged axons were detected in remyelinating lesions, it did not reach significance (Figure 6c). However, these data support the hypothesis that damaged axons can be remyelinated after a demyelinating event, which further substantiates the beneficial role of early remyelination in multiple sclerosis.

### 3.4 Characterization of de‐ and remyelinating and chronic multiple sclerosis lesions

**Figure 7:**
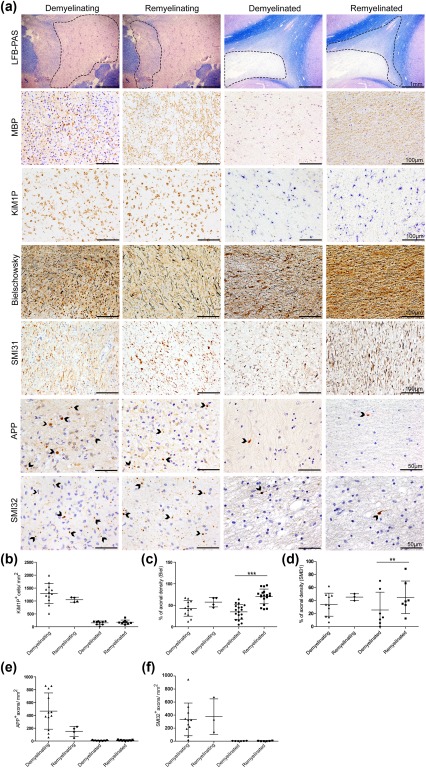
Less axonal loss in remyelinated multiple sclerosis lesions. (a) LFB‐PAS histochemistry shows examples of early (demyelinating and remyelinating) and late stage (demyelinated and remyelinated) multiple sclerosis lesions. (Re)myelination in the lesions was confirmed by immunohistochemistry for myelin basic protein (MBP). (b) Early stage lesions exhibited a higher density of KiM1P^+^ macrophages/activated microglia compared with late stage lesions. (a, c, d) Axonal loss was observed in all lesion stages with a higher axonal density in remyelinated multiple sclerosis lesions. (a) APP^+^ and SMI32^+^ axons reflecting axonal injury were detected in all types of lesions investigated. (e, f) Axonal injury and KiM1P^+^ macrophages/activated microglia occurred more frequently in early than in late stage multiple sclerosis lesions with no significant difference between lesions displaying demyelination and lesions showing remyelination. (c, d) Remyelinated multiple sclerosis lesions revealed a higher axonal density than early stage lesions and demyelinated lesions (** *p* < 0.01; *** *p* < 0.001; *n* = 3‐18; mean ± SD)

The MS lesions studied here showed a confluent actively demyelinating or demyelinated area with or without remyelination. Remyelinated lesions were characterized by reestablished myelin sheaths, which appeared less dense, less organized, and thinner than in adjacent normal appearing white matter in LFB histochemistry (Figure 7a). In addition, early demyelinating and remyelinating lesions exhibited a prominent macrophage infiltration with detection of LFB‐ and MBP‐positive myelin degradation products. In contrast, chronic demyelinated and remyelinated lesions displayed only low numbers of KiM1P^+^ macrophages that were devoid of myelin degradation products (Figure 7a, b).

First, we assessed the amount of damaged axons, that is axons with axonal transport disturbance (APP^+^ axons and spheroids) and axons with dephosphorylated neurofilaments (SMI32^+^ axons). Both were frequently observed in early stage demyelinating as well as remyelinating lesions (Figure 7a, e, f). We did not find a significant difference in the extent of axonal damage in demyelinating vs. early remyelinating lesions and in chronic demyelinated vs. remyelinated lesions. Second, we asked, whether the overall axonal density differs in all investigated multiple sclerosis lesions. The axonal density analyzed in the Bielschowsky silver impregnation and SMI31 immunohistochemistry was comparable in demyelinating and remyelinating lesions, while chronic remyelinated lesions harbored significantly more axons than chronic demyelinated lesions (Figure 7a, c, d). The overall axonal density in early stage lesions was lower than in chronic remyelinated lesions, most likely because of tissue edema and inflammatory cell infiltration. In conclusion, our results support a beneficial role of early and late remyelination.

## 4 DISCUSSION

The precise timing of early remyelination in relation to axonal damage has not yet been studied in detail. In the present study, we performed tight time course experiments in the cuprizone mouse model and in focal lysolecithin‐induced demyelination. We observed that damaged axons are able to gain a new myelin sheath, which may act as a “patch” and may thereby promote axonal recuperation. It has long been assumed that remyelination is beneficial for axonal preservation in multiple sclerosis in the long term (Kornek et al., 2000; Kuhlmann, Lingfeld, Bitsch, Schuchardt, & Brück, 2002; Patrikios et al., 2006), which we confirmed in our study of early and late stage multiple sclerosis lesions. Hence, our data support the notion that therapies promoting remyelination would be beneficial at early and late stages of lesion development to increase the axonal preservation.

After cuprizone challenge the corpus callosum of mice exhibited acutely damaged axons, which were identified as APP^+^ axons or axonal spheroids. APP^+^ axonal profiles may reflect axonal transection or reversible transport disturbance (Nikic et al., 2011). The temporal sequence of axonal swelling and transection as a consequence of axonal damage and the reversibility of acute axonal damage were recently studied in EAE by *in vivo* imaging. This work also showed that APP^+^ axons in the immediate perilesional area of early lesions were in part myelinated, suggesting that acute axonal damage may in part occur independently of demyelination. In the present study, we observed that in the cuprizone as well as lysolecithin models, APP^+^ myelinated axons increased with lesion repair and remyelination. Thus, remyelination of damaged axons, as observed here, may be a possibility to facilitate axonal recuperation. However, the pathological mechanisms in the cuprizone model are different from the autoimmune EAE based on activated encephalitogenic T cells. Only a negligible amount of peripheral immune cells infiltrate the corpus callosum of cuprizone treated mice, while activated and phagocytosing microglia predominate (Hiremath, Chen, Suzuki, Ting, & Matsushima, 2008; Matsushima & Morell, 2001; Mildner et al., 2007). Therefore, the mechanisms of axonal pathology and recuperation might differ as well. However, we also observed remyelination of damaged axons in lysolecithin‐induced focal demyelination, indicating that remyelination of functionally impaired axons is not restricted to cuprizone‐induced demyelination.

In the present study, the number of injured axons decreased continuously during remyelination in the cuprizone model and also in lysolecithin‐induced demyelination. Remyelination thus seemed to be axon‐protective, and no evidence for heightened vulnerability of axons during early remyelination was observed in our models (Irvine & Blakemore, 2006, 2008). In line, previous studies have demonstrated a beneficial effect of remyelination in the cuprizone model by showing increased axonal damage after remyelination failure induced by oligodendrocyte precursor cell depletion, and decreased axonal damage and better axonal preservation after induction of remyelination by neurosphere transplantation (Irvine & Blakemore, 2008). Also, restoration of neurological function could serve as an indicator of increased axon protection. Along this line, Murray and colleagues reported a partial restoration of neurological functions coinciding with spontaneous remyelination in a viral model of multiple sclerosis (Murray, McGavern, Sathornsumetee, & Rodriguez, 2001). Taken together, these results support an axon‐protective role of remyelination substantiating an important role for therapies that promote remyelination.

For most of the studies exploring mechanisms of demyelination, remyelination and axonal damage in the cuprizone mouse model, time intervals of weeks or months were used (Gudi et al., 2009; Lindner, Fokuhl, Linsmeier, Trebst, & Stangel, 2009; Lindner et al., 2008; Skripuletz et al., 2013; Stidworthy, Genoud, Suter, Mantei, & Franklin, 2003). In the present study the very short time intervals of only days enabled a detailed analysis of early remyelination and axonal damage. Acute axonal damage decreased continuously during recovery presumably in part because of rapid and efficient oligodendrocyte differentiation and remyelination. However, acute axonal damage (∼9 APP^+^ axons/mm^2^) was shown to still occur after long‐term remyelination (28 weeks) (Manrique‐Hoyos et al., 2012). The axonal damage, which was evaluated after one (median 163.3 APP^+^ axons/mm^2^) or three (median 129.2 APP^+^ axons/mm^2^) weeks of remyelination in the present work, was much higher than the axonal damage observed in the study of Manrique‐Hoyos and colleagues, indicating that axonal damage is more prevalent early after cuprizone‐diet cessation. Furthermore, damaged or transected axons could be detected by immunohistochemistry for APP already a few hours after injury (McKenzie et al., 1996; Otsuka, Tomonaga, & Ikeda, 1991) and were observed up to four weeks after the insult (Bramlett, Kraydieh, Green, & Dietrich, 1997; Pierce, Trojanowski, Graham, Smith, & McIntosh, 1996). Therefore, the majority of the axonal damage probably occurred during cuprizone treatment and demyelination and was still detectable during the phase of remyelination.

Our study revealed that 25% of APP^+^ axons appeared myelinated already after 7 days of remyelination in the cuprizone model. The temporal relation of axonal damage and early remyelination suggests that axons were efficiently remyelinated independent of their functional impairment. We could also identify APP^+^ myelinated axons in lysolecithin‐induced demyelination in the stage of lesion repair. These data are in line with work on chronically demyelinated axons, which can still be efficiently remyelinated (Foote & Blakemore, 2005; Mason et al., 2004). Thus, it was proposed that efficient remyelination depends rather on the presence of myelinating oligodendrocytes and specific environmental factors than on the axons themselves. Moreover, a functional axonal transport does not seem to be a necessary precondition for the establishment of a proper myelin sheath.

We could demonstrate that the number of myelinated injured axons was increased in early remyelinating multiple sclerosis lesions compared with demyelinating lesions. This observation suggests that a similar sequence of events might lead to myelinated damaged axons in multiple sclerosis. However, the models employed here are devoid of a significant contribution of adaptive immune cells, and primarily suited to investigate mechanisms of de‐ and remyelination. Yet, in multiple sclerosis, the focal and diffuse immune activation, including adaptive and innate immune cells, might influence the process of remyelination and the extent and occurrence of axonal damage.

In conclusion, our work contributes to the elucidation of the mechanisms that are important for axonal preservation and remyelination. The findings of this study demonstrate that axons with functional impairment, including axonal transport disturbances can still be remyelinated. Remyelination may not only restore saltatory conduction, but may also actively assist in the preservation of axonal integrity after axonal damage.

## Supporting information

Supporting Information 1

Supporting Information 2
