# Supplementary material for: Acutely damaged axons are remyelinated in multiple sclerosis and experimental models of demyelination
Source: Glia. 2017 May 31;65(8):1350–60. doi: 10.1002/glia.23167 (PMC5518437; doi:10.1002/glia.23167)
Supplement: Supplementary file 1 — Supporting Information 1 [file GLIA-65-1350-s001.doc]

**Supplementary Table 1: Clinical findings in patients with inflammatory demyelinating biopsies**

| **MS**  **case** | **Sex** | **Age at disease onset (years)** | **Disease**  **duration (months)** | **Disease**  **course** | **Presenting**  **symptom(s)** | **Brain MRI** | **Index lesion** | **OCB** |
| --- | --- | --- | --- | --- | --- | --- | --- | --- |
| #1 | M | 34 | 0.5-0.75 | CIS^*^ | Right hemiparesis, right hemihypesthesia | Multiple intracerebral WM lesions, esp. parietal lobe | Left parietal | + |
| #2 | F | 43 | 23 | RRMS | Walking difficulties, paresthesia, ataxia; progressive; spastic paraparesis, urinary incontinence | Large periventricular hyperintense lesions | Left parietal | n.a. |
| #3 | F | 62 | 0.5-0.75 | CIS (2 ys follow-up) | Disorientation, walking difficulties | Bi-occipital, subcortical and periventricular in part contrast enhancing lesions | Right parietal | - |
| #4 | F | 46 | n.a. | n.a. | n.a. | n.a. | n.a. | n.a. |
| #5 | M | 64 | n.a. | n.a. | n.a. | Cystic, left frontal tumefactive lesion | Left frontal | n.a. |
| #6 | F | 33 | 144 | RRMS | Ataxia, dysarthria, nystagmus, tetraspasticity, incontinence, depression | Multiple supra- and infratentorial WM lesions; large bifrontal, contrast enhancing WM lesion | Right frontal | + |
| #7 | F | 55 | 0.5-0.75 | RRMS | Bilateral paresthesias (feet); paresis left N. VI | Multiple T2 hyperintense lesions, in part contrast enhancing; in part cystic; including the corpus callosum; 2 spinal lesions | Right frontal, cortical | + |
| #8 | F | 23 | 60 | RRMS | Tetraspasticity, ataxia, anarthria, dysphagia, incontinence | Multiple WM lesions brain and spinal cord, in part enhancing | n.a. | + |
| #9 | M | 18 | 120 | SPMS | Paraperesis, gait ataxia, fatique | Multiple supra- and infratentorial WM lesions | n.a. | n.a. |
| #10 | F | 33 | ~2 | RRMS | Left hemiparesis and hemihypesthesia | Multiple, in part enhancing WM lesions, mainly in the right hemisphere | Right frontal | + |
| #11 | F | 30 | 0.4 | RRMS | Sensorimotor hemiparesis | Right periventricular lesion, contrast enhancing, several WM lesions | Right frontal | + |
| #12 | F | 28 | ~0.4 | CIS^*^ | n.a. | Contrast-enhancing periventricular tumefactive lesion with perifocal edema | Right occipital | n.a. |
| #13 | M | 29 | 2 | RRMS | Optic neuritis, hemianopsia | Multiple WM lesions; left and right periventricular lesions (dorsal horn) | Left occipital | + |
| #14 | F | 25 | 4 | RRMS | vertigo, ataxia, hemihypesthesia | Multiple cystic and solid tumefactive lesions; in part perifocal edema, in part ring-enhancing; rapid increase in lesion number | Right frontal | - |
| #15 | F | n.a. | n.a. | CIS^*^ | n.a. | Right fronto-parietal lesion | Right fronto-parietal | n.a. |
| #16 | M | 41 | ~0.4 | CIS^*^ | Seizure, vertigo, hypesthesia both arms and hands, headache | Left frontal tumefactive lesion | Left frontal | n.a. |
| #17 | M | n.a. | ~1 | CIS^*^ | Vertigo, hemihypesthesia, | Parieto-temporal subcortical cystic tumefactive lesion | Left parieto-temporal | n.a. |
| #18 | F | 52 | 0.5 | CIS^*^ | Right hemiparesis | Left parietal, singular contrast-enhancing lesion | Left precentral | - |
| #19 | F | 36 | 12 | RRMS | Memory deficits, disorientation, incomplete internuclear ophthalmoplegia; paraesthesias right leg | Several contrast enhancing WM lesions; spinal lesions | Left parietal | + |
| #20 | M | 57 | ~0.4 | CIS^*^ | Vertigo, instability | Right fronto-temporal periventricular lesion | Right temporal | n.a. |
|  |  |  |  |  |  |  |  |  |

*clinical course at the time point of biopsy. CIS: clinically isolated syndrome - F: female - M: male - MRI: magnetic resonance imaging - MS: multiple sclerosis - n.a.: not available - OCB: oligoclonal bands - RRMS: relapsing remitting multiple sclerosis - SPMS: secondary progressive multiple sclerosis - WM: white matter.
