# Supplementary material for: Acutely damaged axons are remyelinated in multiple sclerosis and experimental models of demyelination
Source: Glia. 2017 May 31;65(8):1350–60. doi: 10.1002/glia.23167 (PMC5518437; doi:10.1002/glia.23167)
Supplement: Supplementary file 2 — Supporting Information 2 [file GLIA-65-1350-s002.doc]

**Supplementary Table 2: Clinical findings in multiple sclerosis autopsies**

| **MS Case** | **Age (years)/Sex** | **Disease duration (years)** | **Disease course** |
| --- | --- | --- | --- |
| 1 | 67/M | >10 | C(P)MS |
| 2 | 55/M | >10 | C(P)MS |
| 3 | 61/F | 18 | C(P)MS |
| 4 | 50/F | >10 | C(P)MS |
| 5 | 28/M | 2,5 | n.a. |
| 6 | 41/M | >10 | C(P)MS |
| 7 | 51/F | 31 | PPMS |
| 8 | 63/F | >10 | C(P)MS |
| 9 | 58/F | >10 | C(P)MS |
| 10 | 49/M | >10 | C(P)MS |
| 11 | 43/F | >10 | C(P)MS |
| 12 | 59/F | 9 | PPMS |

C(P)MS: chronic (progressive) multiple sclerosis - F: Female - M: Male - n.a.: not available - MS: Multiple sclerosis - PPMS: Primary progressive multiple sclerosis - RRMS: Relapsing remitting multiple sclerosis - SPMS: Secondary progressive multiple sclerosis. WM: White matter.
